# Comparative Proteomic Analysis of *Toxoplasma gondii* RH Wild-Type and Four SRS29B (SAG1) Knock-Out Clones Reveals Significant Differences between Individual Strains

**DOI:** 10.3390/ijms241310454

**Published:** 2023-06-21

**Authors:** Kai Pascal Alexander Hänggeli, Andrew Hemphill, Norbert Müller, Manfred Heller, Anne-Christine Uldry, Sophie Braga-Lagache, Joachim Müller, Ghalia Boubaker

**Affiliations:** 1 Department of Infectious Diseases and Pathobiology, Institute of Parasitology, University of Bern, 3012 Bern, Switzerland; kai.haenggeli@unibe.ch (K.P.A.H.); andrew.hemphill@unibe.ch (A.H.); norbert.mueller@unibe.ch (N.M.); 2 Graduate School for Cellular and Biomedical Sciences, University of Bern, 3012 Bern, Switzerland; 3 Proteomics and Mass Spectrometry Core Facility (PMSCF), Department for BioMedical Research (DBMR), University of Bern, 3012 Bern, Switzerland; manfred.heller@unibe.ch (M.H.); pmscf.dbmr@unibe.ch (A.-C.U.);

**Keywords:** genetic manipulation, pleiotropic effects, systems biology

## Abstract

In *T. gondii*, as well as in other model organisms, gene knock-out using CRISPR-Cas9 is a suitable tool to identify the role of specific genes. The general consensus implies that only the gene of interest is affected by the knock-out. Is this really the case? In a previous study, we generated knock-out (KO) clones of TgRH88_077450 (SRS29B; SAG1) which differed in the numbers of the integrated dihydrofolate-reductase-thymidylate-synthase (MDHFR-TS) drug-selectable marker. Clones 18 and 33 had a single insertion of MDHFR-TS within SRS29B. Clone 6 was disrupted by the insertion of a short unrelated DNA-sequence, but the marker was integrated elsewhere. In clone 30, the marker was inserted into SRS29B, and several other MDHFR-TS copies were found in the genome. KO and wild-type (WT) tachyzoites had similar shapes, dimensions, and vitality. This prompted us to investigate the impact of genetic engineering on the overall proteome patterns of the four clones as compared to the respective WT. Comparative shotgun proteomics of the five strains was performed. Overall, 3236 proteins were identified. Principal component analysis of the proteomes revealed five distinct clusters corresponding to the five strains by both iTop3 and iLFQ algorithms. Detailed analysis of the differentially expressed proteins revealed that the target of the KO, srs29B, was lacking in all KO clones. In addition to this protein, 20 other proteins were differentially expressed between KO clones and WT or between different KO clones. The protein exhibiting the highest variation between the five strains was srs36D encoded by TgRH_016110. The deregulated expression of SRS36D was further validated by quantitative PCR. Moreover, the transcript levels of three other selected SRS genes, namely SRS36B, SRS46, and SRS57, exhibited significant differences between individual strains. These results indicate that knocking out a given gene may affect the expression of other genes. Therefore, care must be taken when specific phenotypes are regarded as a direct consequence of the KO of a given gene.

## 1. Introduction

Human toxoplasmosis, caused by the apicomplexan parasite *Toxoplasma gondii*, is one of the most prevalent parasitic diseases, with one-third of the human population on Earth being chronically infected [1,2]. In Europe and North America, three main genotypes (I-III) prevail [3], but new and atypical genotypes, distinct from I-III, have been discovered, most notably in South America [4].

For over three decades, *T. gondii* has been exploited as an excellent molecular genetic model to investigate intracellular apicomplexan parasites [5]. *T. gondii* ME49, a type II strain, has been the first of several strains whose genomes have been sequenced (toxodb.org), and CRISPR-CAS-mediated genome engineering [6] has led to the identification of a large number of fitness-conferring genes [7]. It is widely assumed that the inactivation of a specific gene can cause a reproducible and identical impact on parasite biology, regardless of the strain or the genetic modification methods employed. This is largely evidenced by the application of a single comparison frame, “one modified clone versus wild-type parasites,” in many reports.

However, since genetic engineering is prone to interfere with fundamental cellular functions, it is worth investigating which impact this procedure has on the systems biology of the organism. A suitable strategy to investigate such effects is the analysis of differentially expressed proteins in transformed clones and respective non-transformed wild-type (WT) parasites. For instance, in *Giardia lamblia*, nearly 10% of the overall proteome is altered upon transformation with a stable plasmid [8]. In order to investigate to what degree protein expression patterns other than the intended knockout (KO) are affected in *T. gondii*, a non-essential gene should be chosen whose KO has no impact on fitness, at least not during the in vitro culture of tachyzoites.

*T. gondii*, as well as other apicomplexan protozoans, express a panoply of surface proteins involved in many aspects of host–parasite interactions [9]. The surface antigen 1 (sag1), sometimes designated as p29 or p30, is the predominant surface antigen of *T. gondii* [10]. It is a member of a family of closely related proteins called “sag-related sequence proteins (srs proteins)”, as evidenced three decades ago [11,12]. The first genomic sequencing efforts revealed the existence of a family of 161 SRS genes with two subfamilies, with SAG1 and SAG2A as prototypic members in the Me49 strain [13]. Later, this number was corrected to 109 SRS genes [14]. These genes are designated as SRS followed by a number. For instance, SAG1 is named SRS29B, and SAG2A became SRS34A. Proteins encoded by SRS genes have from one to four cysteine-rich SRS protein domains and a c-terminal glycosyl-phosphatidyl-inositol (GPI) anchor domain providing a flexible attachment on the parasite surface [14]. SRS29B Kos generated earlier by chemical mutagenesis [15], or more recently by genetic engineering [6,10,14], do not exhibit marked effects on proliferation during in vitro culture. In rodent models, however, interactions with the immune system are altered [10,14]. Thus, SRS29B is a suitable candidate to test our hypothesis that generating a KO clone impacts protein expression patterns other than the intended KO target protein limiting indirect effects linked, e.g., to growth impairment.

In a previous study, using CRISPR-Cas9, we generated four SRS29B KO clones in the type I strain *T. gondii* RH which displayed different integration patterns of the dihydrofolate reductase-thymidylate synthase (MDHFR-TS) selection marker [16]. The clones differed regarding the copy numbers and the mdhfr-ts insertion pattern. Clones 18 and clone 33 both had a single-copy MDHFR-TS insertion within SRS29B. Clone 6 also had a single-copy MDHFR-TS insertion in the genome, although not within SRS29B. Instead, SRS29B was disrupted by the insertion of a short DNA sequence unrelated to the resistance marker. Clone 30 exhibited multiple copies of MDHFR-TS in the genome, with one copy disrupting SRS29B [16]. In the present study, we compare the proteome patterns of these four clones to their respective WT *T. gondii* RH using whole-cell shotgun LC-MS/MS and show that—besides the intended KO—the expression patterns of other proteins are altered.

## 2. Results

**Figure 1:**
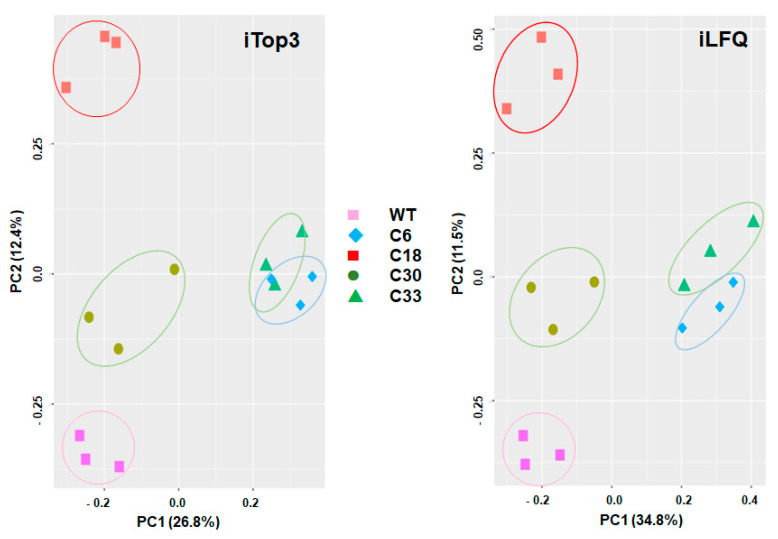
Principal component analysis of proteome data set of TgRH_SRS29B KO clones and their corresponding WT Tg RH. Whole-cell shotgun LC-MS/MS and analysis of the data were performed as described in Section 4. Principal components of the iTop3 (**left panel**) and iLFQ (**right panel**) datasets are presented. For each strain, three biological replicates are shown. C, clone; PC, principal component; WT, wild type.

Differential proteomic analysis of the Tg_SRS29B KO clones versus their corresponding *T. gondii* RH wild type yielded 36,476 unique peptides matching 3236 *T. gondii* proteins. The complete dataset is available as supplemental Table S1. Overall analysis of the data by principal component analysis revealed four non-overlapping clusters of the biological replicates of WT, clone6/clone33, clone18, and clone 30 by both iLFQ and iTop3 algorithms. The biological replicates of clones 6 and 33 were closer to each other than to other strains (Figure 1).

A more detailed analysis revealed that 21 proteins were significantly downregulated between the WT and the KO clones or between the different KO clones, taking into account both Top3 and LFQ algorithms to enhance belief in our results.

**Figure 2:**
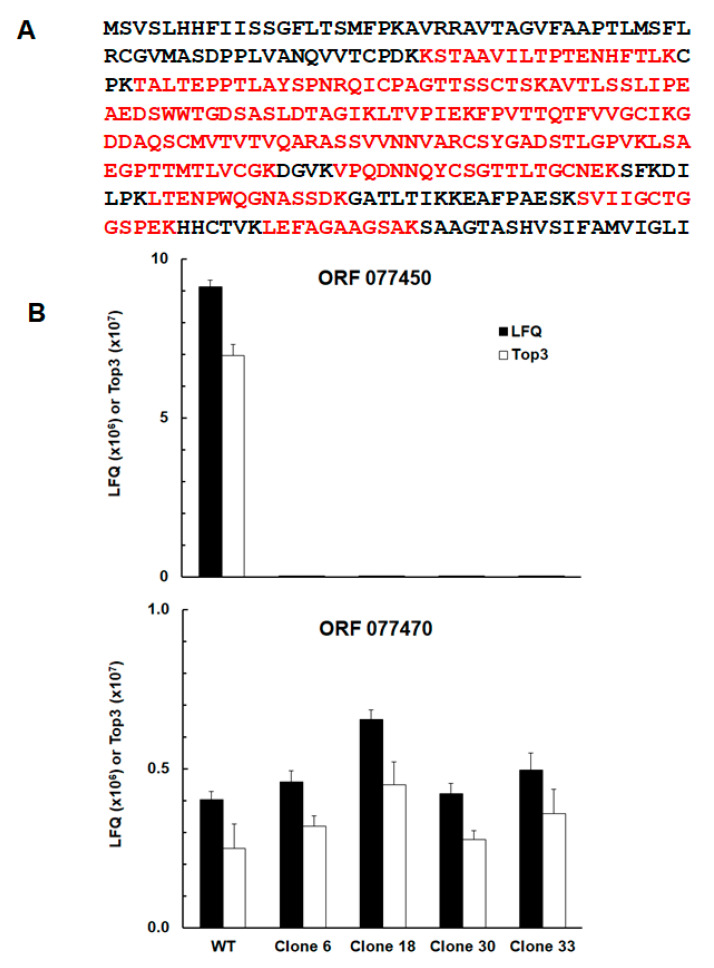
The protein srs29B encoded by open reading frame (ORF) TGRH88_077450 is lacking in the respective KO clones. (**A**) Protein sequence of srs29B with peptides detected in WT samples highlighted in red. (**B**) LFQ (black) and Top3 (white) values corresponding to ORF 077450 and ORF 077470 (srs29c). Whole-cell shotgun LC-MS/MS and analysis of the data were performed as described in Section 4. Mean values ± standard deviation correspond to three biological replicates.

Peptides belonging to protein srs29B or sag1 encoded by the open reading frame (ORF) TgRH88_077450 (Figure 2A) were unambiguously detected in WT tachyzoites only. In three of the KO clones, C6, C18, and C33, sag1 peptides were detected in trace amounts (ca. three orders of magnitude lower than in the WT) in one of three biological replicates only. In clone 33, sag1 peptides were not detectable (Figure 2B; Table S1). The protein with the highest identity to srs29B, the syntenic srs29C encoded by TgRH88_077470 (E value 6 × 10^−31^) did not significantly differ between WT and KO clones (Figure 2B).

Altered expression levels were detected in KO clones not only for srs29B, the target protein of the KO, but also for 15 other proteins (Table 1). Eight of these proteins were differentially expressed between WT and KO clones only, and six proteins were also differentially expressed between individual KO clones. None of these proteins were differential in all KO clones as compared to the WT (Table 1). 

Moreover, six proteins were differential between different TgRH_SRS29B KO clones only. These proteins are listed in Table 2.

**Figure 3:**
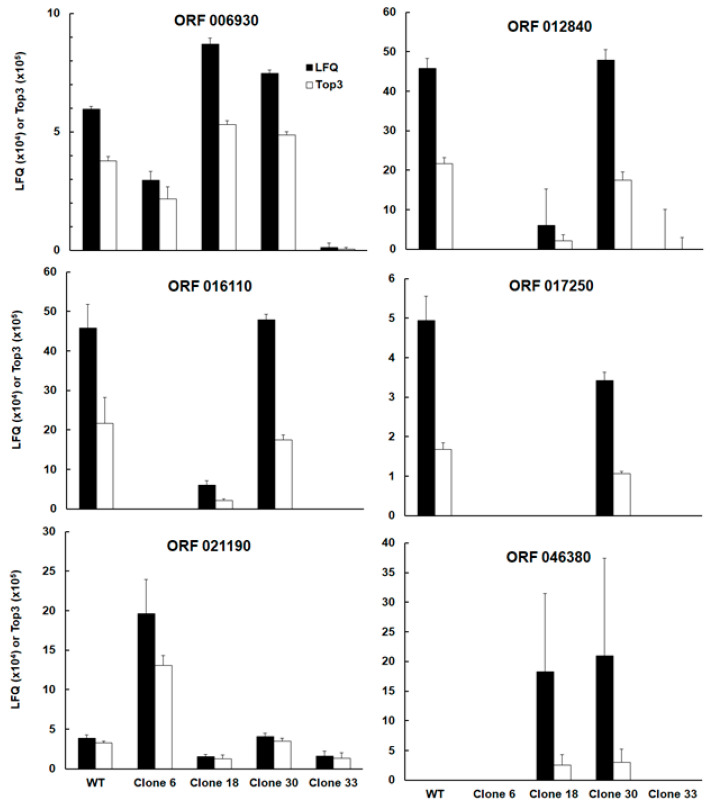
Abundances of the six proteins with differential expression between WT and KO clones designated by their corresponding TgRH88 ORFs. Annotations and significant differences between strains are indicated in Table 1 and Table 2. Whole-cell shotgun LC-MS/MS and analysis of the data were performed as described in Section 4. LFQ (black) and Top3 (white) mean values ± standard deviation correspond to three biological replicates.

Interestingly, the list of differentially expressed proteins comprised—besides the intended KO SRS29B—two other SAG-related sequences, namely srs16C, encoded by the ORF TgRH88_006930, and srs36D, encoded by TgRH88_016110. Note that srs16C had significantly lower abundances in clone 33 as compared to the WT, clones 18, and clone 30; moreover, srs36D was equally abundant in wild-type and clone 30 and had significantly lower expression levels in the other clones. The abundances srs36D and srs16C in WT tachyzoites were one and two magnitudes lower, respectively, compared to the abundance of srs2929B (Figure 3; ORF 006930, ORF 016110).

Among the four other proteins with differential expression between WT and KO clones and between clones was the protein encoded by TGRH88_012840. Annotated as an unspecific product in the TgRH88 database and as a putative transmembrane protein in other strains, this protein had equal abundances in WT and clone 30 and exhibited lower levels in the other clones (Figure 3; ORF 012840). Due to high variations between biological replicates, the expression levels of ORF 012840 were significantly different only between WT and clone 6, and between clone 30 and clone 6, respectively, while clone 18 expression levels exhibited no significant difference to the WT. This protein was expressed in similar amounts as srs36D in WT and clone 30 tachyzoites (Figure 3; ORF 012840). Another protein with similar levels in WT and clone 30 was the protein encoded by ORF 017250, while in the three other clones, this protein was not detectable. Its expression levels in WT and clone 30 tachyzoites was in the same range as seen for srs16C (Figure 3; ORF 017250). The protein encoded by ORF 004470 had lower levels in clone 33 as compared to WT and the other clones (Figure 3; ORF 004470). Another expression pattern was observed with the protein encoded by TgRH_021190, a cAMP-dependent protein kinase. This protein was expressed at significantly higher levels in clone 6 than in WT tachyzoites and the other clones (Figure 3; ORF 021190). Finally, the protein encoded by ORF 046380, a histone lysine methyltransferase set1 homolog, could be detected in tachyzoites from clones 18 and 30 only.

**Figure 4:**
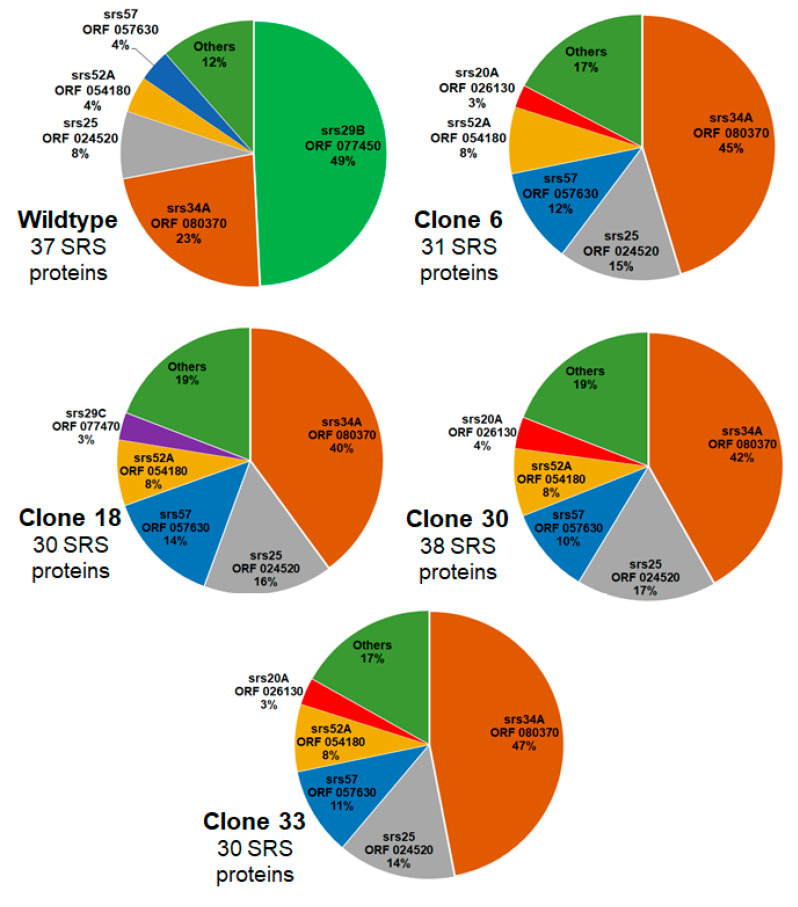
Relative abundances of SRS proteins in WT and KO clones designed by their names and the corresponding TgRH88 ORFs. Whole-cell shotgun LC-MS/MS and analysis of the data were performed as described in Section 4. The percentage values were calculated based on the iBAQ values of the respective proteins as listed in Table S2, with 100% being the total amount of SRS proteins for each strain. The numbers correspond to SRS proteins detected in at least two biological replicates in each strain.

Since SRS proteins other than sag1 were differentially expressed in the KO clones, we investigated the relative abundance of SRS proteins in WT and KO clones. In WT tachyzoites, 37 SRS proteins were detectable in at least two biological replicates, and srs29B (sag1, encoded by ORF 077450) was the most abundant one, with nearly 50% of SRS proteins being srs29B. The second most abundant SRS protein was srs34A (23%; encoded by ORF 080370), followed by srs25 (encoded by ORF 024520), srs52A (ORF 054180), and srs57 (057630). The remaining 33 SRS proteins amounted to 12%. In all KO clones, srs34A (sag2A) became the most abundant SRS protein, representing between 40% and 47% of total SRS proteins in clone 18 and 33, respectively. The second most abundant SRS protein in these KO clones was srs25, followed by srs57 and srs52A as third and fourth most abundant SRS proteins in all clones. In clone 18, srs29C (ORF 077470) was the fifth most abundant SRS protein; in all other clones, srs20A (ORF 026130) took this place. A schematic overview of the relative abundances based on iBAQ values of the proteins is presented as pie charts in Figure 4, and the corresponding data are given as supplemental Table S2. A complementary presentation of the relative abundances of the major SRS proteins in all strains is shown in Table S2 (second page). 

The respective relative abundances of the differentially expressed srs16C (ORF 006930) were 0.1% in WT and clone 6, 0.3% in clones 18 and 33, and less than 0.01% in clone 33. The relative abundance of srs36D (ORF 016110) in WT tachyzoites was one magnitude higher than the abundance of srs16C, amounting to 1%. This protein was equally abundant in clone 30 with 1.5% of total SRS proteins. In clone 18, it represented only 0.1% and was not detectable in clone 6 and clone 33 tachyzoites (Table S2). 

**Figure 5:**
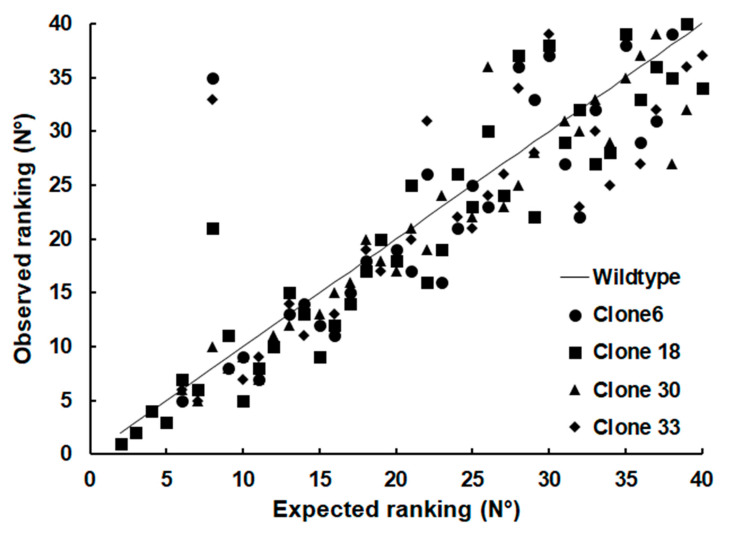
Expected vs. observed ranking of relative abundances of SRS proteins in tachyzoites of WT and SRS29B KO clones. Whole-cell shotgun LC-MS/MS and analysis of the data were performed as described in Section 4. The relative abundances were calculated based on the iBAQ values of the respective proteins and ranked from 1 (the most abundant protein within a class) to n (the least abundant protein). The values are listed in Table S2. The expected ranking was the value of the WT (black line). To avoid distortions, Sag1 (srs29B; ORF 077450), the most abundant SRS protein in the wild type, was omitted from the plot. Therefore, the plot of the WT ranking starts with 2. Without variation in rankings, all points together would form a straight line.

When it comes to less abundant SRS proteins, however, the difference of their relative abundances (see Table S2) between the strains became clearly visible, as visualized by plotting the ranking of the relative abundances against the ranking of the WT proteins (Figure 5).

**Figure 6:**
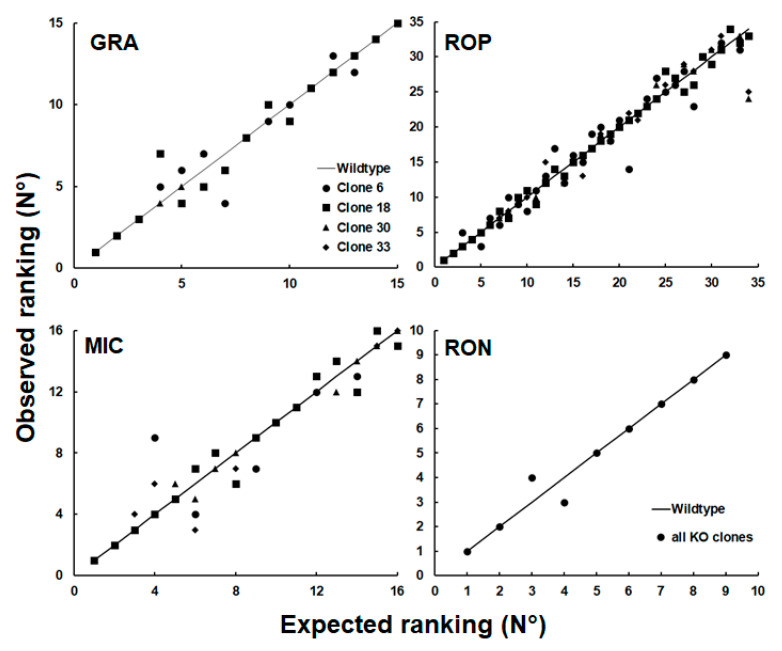
Expected vs. observed ranking of relative abundances of members of different classes of proteins of WT and SRS29B KO clones. Whole-cell shotgun LC-MS/MS and analysis of the data were performed as described in Section 4. The relative abundances were calculated based on the iBAQ values of the respective proteins and ranked from 1 (the most abundant protein within a class) to n (the least abundant protein). The values are listed in Table S3. The expected ranking was the value of the WT (black line). GRA, dense granule proteins; MIC, microneme proteins; ROP, rhoptry proteins; RON, rhoptry neck proteins. Without variation in rankings, all points together would form a straight line.

To investigate whether this observation could be generalized to other groups of proteins, we plotted the relative abundance rankings of four other classes of excretory/secretory proteins, namely dense granule proteins (GRA; 15 proteins detected), microneme proteins (MIC; 16 proteins), rhoptry proteins (ROP; 34 proteins), and rhoptry neck proteins (RON; 9 proteins), based on the dataset presented in Table S3. As shown in Figure 6 the rankings of proteins of these groups were more homogeneous, with only one permutation in the RON group. 

**Figure 7:**
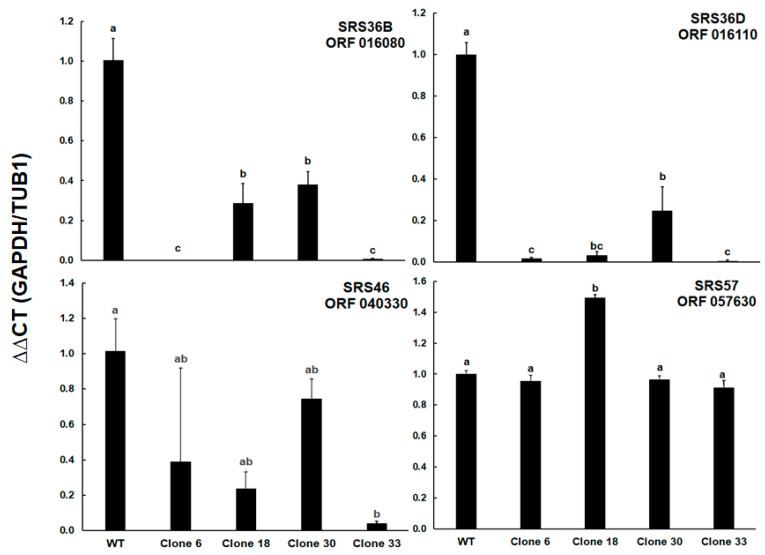
mRNA expression levels of genes encoding four selected SRS proteins. Transcript levels were determined by multiplex TaqMan—qPCR. Bars represents the mean of three biological replicates ± standard deviation. Values superscribed by the same letters are not significantly different from each other (ANOVA, followed by multiple *t*-tests; *p* < 0.05 after Bonferroni adjustment). The complete dataset including exact *p*-values is presented as Table S4.

These results prompted us to determine the relative levels of transcripts of ORFs encoding four selected SRS proteins. In addition to ORF 016110 encoding the differentially expressed srs36D, ORF 016080 encoding srs36B (sag9) was included. This SRS protein was expressed not only in tachyzoites, but also in bradyzoites [17] of type II and III strains (profiles in ToxoDB). Furthermore, ORF 040330 encoding srs46 with constant expression levels in cell cultures (profiles in ToxoDB), and ORF 057630 encoding srs57 (sag3), one of the more abundant SRS proteins in all strains and an important virulence factor [18], were included. The full dataset is presented as supplemental Table S4. However, the mRNA levels of ORF 057630 were similar among all clones except for clone 18, where the transcript levels were 1.5-times higher than in the other strains. Both ORFs 0161080 and 016110 had significantly lower transcript levels in all KO clones as compared to the wild type. In the case of ORF 016080, clones 6 and 33 had significantly lower levels than clones 18 and 30; in the case of ORF 016110, the transcript levels in these two clones were significantly lower compared to clone 30 only. Significantly lower transcript levels were also noted for ORF 040330 between WT and clone 33 tachyzoites (Figure 7).

## 3. Discussion

Overall, upon the application of very strict criteria for determining differential expression levels, SRS29B KO clones and WT tachyzoites varied by 21 out of 3236 proteins, which corresponds to 0.65%. This variability is far below the nearly 10% of differentially expressed proteins detected in WT and transfected clones of the phylogenetically distant protist *Giardia lamblia*, for which the same strict criteria were applied [8]. However, as illustrated by the principal component analysis plots, the herein presented datasets on *T. gondii* WT and KO clones did not simply form two clusters of *T. gondii* WT proteome and KO proteome. Instead, WT and each KO clone clustered separately, with clone 6 (SRS29B disrupted by a short sequence) and 33 (SRS29B disrupted by resistance marker) closer together than the other clones. The proteomes from KO clones 18 and 30 (SRS29B disrupted by resistance marker, but multiple insertions of the resistance marker in the genome of clone 30) were separated from the WT proteomes by one principal component only. Conversely, the proteomes of clone 33 (with a single integration of the resistance marker into SRS29b and supposedly similar to clone 18 in terms of targeted SRS29B gene editing [16]) and of clone 6 were separated from the clone 18 and WT proteomes by both principal components. A potential factor involved in these proteomic differences among seemingly identical SRS29B KO clones could be the high and divergent phenotypic plasticity of *T. gondii*, in particular with respect to parasite–host interaction [19,20]. In particular, expression levels of SRS genes are highly variable, as shown by transcript analyses of subclones of a given parental strain during 10 division cycles [21]. Therefore, it is not surprising to find—besides the intended KO of srs29B—five other SRS proteins as well as five putative transmembrane proteins within the set of differentially expressed proteins. Regarding the complete set of SRS gene products identified in the proteomes, we observed differences in numbers of unambiguously identified proteins (30 to 38 depending on the strains), as well as fluctuations of the relative abundances of these proteins between the strains. These fluctuations are less pronounced in other groups of proteins involved in host–parasite interactions. Transcript analyses of four selected ORFs encoding SRS proteins revealing differences between individual KO and WT tachyzoites point toward the same direction. In a previous study, double SAG1-SAG2-KOs were followed by the upregulation of SRS29C expression, resulting in a reduction of virulence in mice [14]. In our single KOs, however, srs29C protein levels were not significantly affected.

Interestingly, variable expression of membrane proteins as a response to various biotic and abiotic stresses is a well-known phenomenon in the intestinal protozoan *G. lamblia* (see for review [22] and [8,23,24] for more recent proteomic investigations) and in *Trypanosoma* sp. [25,26] (both superkingdom Excavata), and may thus be a more general phenomenon among eukaryotes. These effects might be the result of epigenetic effects such as histone acetylation or methylation [27,28,29]. The fact that a histone methyltransferase homolog is among the differentially expressed protein in our dataset underlines this hypothesis. Moreover, genomic DNA modifications may result in antigenic variations. In *Trypanosoma brucei*, antigenic variation is triggered by DNA breaks and recombination events [30,31]. The variability among KO clones of the same gene seen is not restricted to protozoans. It has been shown that KO and WT mice exhibit variabilities in gene expression far beyond the intended KOs [32]. Finally, clonal variation resulting from the selection procedures necessary for obtaining the desired transgenic cell lines may be responsible for the observed variability in gene expression and proteome composition [33,34].

Taken together, our data—in alignment with previously published findings—show that genetic modifications may result in pleiotropic effects in the resulting cell lines. To correlate the affected gene locus (e.g., via KO) and the function of the corresponding gene, the problematics of genetic compensation and adaptability should be considered. If phenotypical analyses are performed (if intended), add-backs should be performed not only with one, but several KO clones, and their respective proteomes and respective transcript profiles should be analyzed.

## 4. Materials and Methods

### 4.1. Chemicals

Unless stated otherwise, all tissue culture media were purchased from Gibco-BRL (Zürich, Switzerland), and biochemicals from Sigma (St. Louis, MO, USA). Primers for real-time PCR (RT-PCR) were purchased from Eurofins (Luxemburg).

### 4.2. Host Cell Culture, Maintenance, and Purification of Tachyzoites

Tachyzoites of *T. gondii* RH WT and the four SRS29B KO clones were maintained in vitro in human foreskin fibroblasts (HFF; PCS-201-010™) as previously described [35].

For analyses of the proteomes, T75 cell culture flasks were seeded with HFF (2 × 10^6^) and cultured at 37 °C/5% of CO_2_. When approximately 80% confluent, HFF monolayers were infected with 1 × 10^6^ tachyzoites. After 72 h of culture, heavily infected monolayers were scraped, syringe lysed with a 25G needle, and filtered through a 47 mm diameter polycarbonate disc filter membrane (pore size 3 µm). The resulting tachyzoite suspensions were centrifuged (15 min, 1000× *g*, 4 °C). To remove the residual media, pellets were washed twice with PBS, and parasites were stored as pellets at −80 °C (5 × 10^8^ tachyzoites per pellet).

### 4.3. Proteomics

Cell pellets were lysed in 100 μL 8M urea/100 mM Tris/HCl pH 8, reduced with 10 mM DTT for 30 min at 37 °C, alkylated with 50 mM iodoacetamide for 30 min in the dark, and proteins precipitated with 5 volumes of acetone at −20 °C overnight. The pellets were suspended in 8M urea in 50 mM Tris/HCl pH 8 to an estimated protein concentration of 2 mg/mL. Effective protein concentration was determined with the Qubit Protein Assay (Invitrogen). The urea concentration was reduced to 1.6 M by dilution with 20 mM Tris/HCl and 2 mM CaCl_2_, and an aliquot corresponding to 10 μg protein was digested with sequencing-grade trypsin (Promega) at room temperature overnight at a protein-to-protease ratio (*w*/*w*) of 50:1. Digestions were stopped with TFA at a final concentration of 1% (*v*/*v*).

The digests were analyzed by a nano-liquid chromatography mass spectrometry system consisting of an Ultimate 3000 (ThermoFischer Scientific, Reinach, Switzerland) coupled to a timsTOF Pro (Bruker Daltonics, Bremen, Germany) through a CaptiveSpray source (Bruker, Bremen, Germany) with an endplate offset of 500 V, a drying temperature of 200 °C, and with the capillary voltage fixed at 1.6 kV. A volume of 2 µL (200 ng) from the protein digest was loaded onto a pre-column (C18 PepMap 100, 5 µm, 100 A, 300 µm i.d. × 5mm length, ThermoFisher) at a flow rate of 10 µL/min with 0.05% TFA in water/acetonitrile 98:2. After loading, peptides were eluted in back flush mode onto a homemade C18 CSH Waters column (1.7 μm, 130 Å, 75 μm × 20 cm) by applying a 90 min gradient of 5% acetonitrile to 40% in water/0.1% formic acid, at a flow rate of 250 nL/min. The TimsTOF Pro was operated in data-dependent acquisition mode using Parallel Acquisition SErial Fragmentation (PASEF). The mass range was set between 100 and 1700 *m*/*z*, with 10 PASEF scans in an ion mobility window of 0.6 and 1.6 V s/cm^2^. The accumulation time was set to 2 ms, and the ramp time was set to 100 ms. Fragmentation was triggered at 20,000 arbitrary units (au), and peptides (up to charge 5) were fragmented using collision-induced dissociation with energies set between 20 and 59 eV dependent on peptide precursor mass.

Mass spectrometry data were processed by MaxQuant [36] software, version 2.0.1.0, against the ToxoDB-52_TgondiiRH88_AnnotatedProteins and a contaminants fasta protein sequence database with matching between runs option using a matching time window of 0.7 min and an ion mobility window of 0.05 1/K0, and giving each cell line a non-consecutive fraction number in order to prevent overinterpretation, respectively. The strict trypsin cleavage rule was applied, allowing for up to three missed cleavages, variable modifications of protein N-terminal acetylation and oxidation of methionine, and static modification of cysteine with carbamidomethylation. Precursor and fragment mass tolerances were set to 20 ppm. Peptide spectrum matches, as well as peptide and protein group identifications, were filtered to a 1% false discovery rate (FDR) based on reversed database sequence matches, and a minimum of two razor or unique peptides were required to accept a protein group identification. MaxQuant’s Intensity-Based Absolute Quantification (iBAQ) values were used to calculate relative abundance by equalizing their sum in each sample. The comparison of protein abundance between groups was made using both MaxQuant’s Label-Free Quantification (LFQ) values as well as Top3 values (sum of the 3 most intense peptide form intensities), as reported elsewhere [7,36]. Protein identifications from the contaminants database (e.g., trypsin or BSA) as well as proteins identified only by site were removed for statistical validation.

### 4.4. Quantification of Transcripts by Multiplex TaqMan—qPCR

Total RNA was extracted from tachyzoites using the NucleoSpin RNA isolation kit (Macherey-Nagel, Duren, Germany). Furthermore, 1 μg of total RNA template was reverse transcribed to cDNA by the GoScript Reverse Transcription System (Promega, Amriswil, Switzerland) using a random hexamer primer under the following conditions: 5 min at 25 °C, 60 min at 42 °C, and 15 min at 70 °C; reactions were then cooled down to 4 °C. All qPCRs were performed in a Bio-Rad CFX 96 QPCR instrument (Biorad) using the FastStart Essential DNA Probes Master (Roche, Basel, Switzerland). The total volume for qPCR was 10 μL, consisting of 5 μL of 1x SensiFast master mix (Bioline, Meridian Bioscience, Cincinnati, OH, USA), 0.5 μM of reverse and forward primers, 0.1 μM of each probe, 0.3 mM of dUTP, one unit of heat-labile Uracil DNA Glycosylase (UDG), and 2.8 μL of cDNA template (diluted 1:5). The primers and probes for four ORFs encoding SRS proteins and for two reference genes are listed in Table 3.

Quantitative PCRs were performed according to the following thermal profile: (1) initial incubation of 10 min at 42 °C, followed by (2) denaturation step of 5min at 95 °C and (3) 50 cycles of two-step amplification (10 s at 95 °C and 20 s at 62 °C). A negative control with double-distilled water was included for each experiment. Relative mRNA expression using multiple reference genes was determined as previously described [37,38] and presented as weighed data with values obtained from wild type tachyzoites arbitrarily set as one.

### 4.5. Statistics

Evaluations of both proteomic and gene expression data were performed based on three biological replicates per individual strain. Strains were compared with each other and not KO strains on one side, while strains were compared with wild type on the other side.

As in previously published studies [8,39], to enhance belief in our data, only proteins which were found differential between the strains by both LFQ and Top3 intensities were regarded as differentially expressed. Briefly [36], peptide forms were normalized by variance stabilization and imputed at this level to form the reported iTop3 values, whereas LFQ values were imputed at the protein group level to form the reported iLFQ values. In both cases, missing values were replaced by the maximum likelihood estimation method if there was more than one identification in the group of replicates. All other missing values were replaced by a random number from a Gaussian distribution of width 0.3 × sample standard deviation and centered at the sample distribution mean minus 2.8 × or 2.5 × sample standard deviation, at peptide form and protein group level, respectively. Differential expression was tested with the moderated *t*-test, and *p*-values were corrected for multiple testing by the Benjamini–Hochberg method. A significance curve was calculated such that a minimum log2 fold change of 1 (absolute value) was required, as well as a maximum adjusted *p*-value of 0.05 as an asymptotically high log2 fold change. Twenty cycles of imputation were performed, and protein groups consistently recorded as differentially expressed were flagged as protein groups of interest. Relative mRNA expression data were analyzed by ANOVA followed by multiple *t*-tests using the software package R (version 1.1.456) [40]. After Bonferroni adjustment, *p*-values of less than 0.05 were considered to be statistically significant.

## Figures and Tables

**Table 1:** List of proteins differentially expressed (DE) between TgRH_SRS29B KO clones and their corresponding WT TgRH. Whole-cell shotgun LC-MS/MS and analysis of the data were performed as described in Section 4. Only proteins exhibiting significantly different expression levels by both Top3 and LFQ were considered. “WT/C up” indicates that the DE protein is significantly higher expressed in the WT than in the respective clone, while “down” indicates lower expression levels of DE in the WT. DE proteins with different levels also between clones are highlighted in italics.

GN	Annotation	WT/C6	WT/C18	WT/C30	WT/C33
TGRH88_003360	Unspecified product (putative transmembrane protein)	down		down	
*TGRH88_006930*	*SAG-related sequence srs16C*				*up*
*TGRH88_012840*	*Unspecified product (putative transmembrane protein)*	*up*			
*TGRH88_016110*	*SAG-related sequence srs36D*	*up*	*up*		*up*
*TGRH88_017250*	*Unspecified product*	*up*	*up*		*up*
*TGRH88_021190*	*cAMP-dependent protein kinase*	*down*			
TGRH88_029080	Gamma-glutamyl hydrolase	up			
TGRH88_029100	Unspecified product	down			
TGRH88_036500	Multi-pass transmembrane protein	down			down
TGRH88_042260	Unspecified product (putative transmembrane protein)	up			up
TGRH88_043760	Putative translation elongation and release factors (GTPase)	down	down		
*TGRH88_046380*	*Histone lysine methyltransferase set1*		*down*	*down*	
TGRH88_055240	Putative myosin heavy chain	up			
TGRH88_062920	Bifunctional dihydrofolate reductase—thymidylate synthase		down		down
TGRH88_073870	MaoC family domain-containing protein	up			
TGRH88_077450	SAG-related sequence srs29B	up	up	up	up

**Table 2:** List of proteins differentially expressed (DE) between TgRH_SRS29B KO clones. Whole-cell shotgun LC-MS/MS and analysis of the data were performed as described in Section 4. Only proteins with significantly different levels by both Top3 and LFQ were considered as DE. ‘X/Y up’ indicates a DE protein level significantly higher in X than in Y. DE proteins with different levels also between clones are highlighted in italics.

GN	Annotation	C6/C18	C6/C30	C6/C33	C18/C30	C18/C33	C30/C33
TGRH88_004120	PRELI family protein					up	
*TGRH88_006930*	*SAG-related sequence srs16C*					*up*	*up*
TGRH88_008350	Unspecified product (putative iron-sulfur cluster assembly accessory protein)	down	down				
TGRH88_009830	SAG-related sequence srs19F	down					
*TGRH88_012840*	*Unspecified product* *(Putative transmembrane protein)*	*down*	*down*				
TGRH88_014370	Cathepsin cpc1	down				up	
*TGRH88_016110*	*SAG-related sequence srs36D*	*down*	*down*		*down*		*up*
TGRH88_016870	Unspecified product			up			up
*TGRH88_017250*	*Unspecified product*		*down*		*down*		
*TGRH88_021190*	*cAMP-dependent protein kinase*	*up*	*up*	*up*			
*TGRH88_046380*	*Histone lysine methyltransferase set1*	*down*	*down*				
TGRH88_076630	Unspecified product	down					

**Table 3:** List of primers and probes for TaqMan-quantitative reverse transcriptase PCR used in this study. BHQ, black hole quencher; Cy5, cyanine 5; FAM, 6-carboxyfluorescein; HEX, hexachlorofluorescein.

Gene ID (ToxoDB)	Gene Name	Forward Primer (5′-3′)	Reverse Primer (5′-3′)	Taqman-Hydrolysis Probe
TGRH88_014440	Glyceraldehyde-3-phosphate dehydro-genase GAPDH1	TGGAGGTTTTGGCGAT	ATGGCAGTTGGCTCCTT	HEX-TACCCCGGC-GAAGTCAGC-BHQ
TGRH88_016080	SAG-related sequence SRS36B (SAG 5D)	TCAGAGGTCACCCGAGT	TCGTGGTGTCCTGGTTAC	FAM-CACCATCCAG-GTGATCCAGCC-BHQ
TGRH88_016110	SAG-related sequence SRS36D (SAG 5C)	TGTATGGCAAGCGACAA	TGTGCACACCTCAGTTAGTG	FAM-ACGGGCAG-AGTGACAGCGCA-BHQ
TGRH88_040330	SAG-related sequence SRS46	TGCAGACGTACCGACAGT	TCTGCGTCGACGAGTG	FAM-TCTTCGCTG-CGGCAAAAACTT-BHQ
TGRH88_055140	Alpha tubulin TUBA1	GACGCCTTCAACACCTTC	TTGTTCGCAGCATCCT	Cy5-TTACCGCCA-CCTGTTCCACCC-BHQ
TGRH88_057630	SAG-related sequence SRS57 (SAG 3)	TGCGATCTTGGGAAC	TCAGGGTGCTCTTTGTTC	FAM-TGTTCGTCG-CCGCAGGGA-BHQ

## Data Availability

All datasets are available as supplementary materials (see above).

## Supplementary Materials

The following supporting information can be downloaded at: https://www.mdpi.com/article/10.3390/ijms241310454/s1.
